# Prediction of Mortality in Hemodialysis Patients Using Inflammation- and Nutrition-Based Indices

**DOI:** 10.3390/jpm15100489

**Published:** 2025-10-13

**Authors:** Umit Cakmak, Nurgul Sevimli, Suleyman Akkaya, Ozgur Merhametsiz

**Affiliations:** 1 Antalya Memorial Hospital, Nephrology Clinic, 07025 Antalya, Turkey; 2 Department of Immunology and Allergy, University of Health Sciences, Gazi Yasargil Training and Research Hospital, 42130 Diyarbakir, Turkey; nurgulsevimli@hotmail.com; 3 Department of Cardiology, University of Health Sciences, Gazi Yasargil Training and Research Hospital, 42130 Diyarbakir, Turkey; slymnkky1982@gmail.com; 4 Nephrology Department, Faculty of Medicine, Yeni Yuzyil University, Private Gaziosmanpasa Hospital, 34245 Istanbul, Turkey

**Keywords:** CRP–albumin–lymphocyte (CALLY) index, hemodialysis, peritoneal dialysis, mortality

## Abstract

**Background and Objectives:** Chronic kidney disease (CKD) and end-stage kidney disease (ESKD) are characterized by persistent inflammation, malnutrition, and immune dysfunction, all of which contribute to poor outcomes in hemodialysis (HD) patients. The C-reactive protein albumin lymphocyte (CALLY) index has been proposed as a novel biomarker that integrates these mechanisms. This study aimed to evaluate the prognostic value of the CALLY index together with established markers, including the C-reactive protein-to-albumin ratio (CAR), neutrophil-to-lymphocyte ratio (NLR), and monocyte-to-lymphocyte ratio (MLR) for all-cause mortality in HD patients. **Materials and Methods:** This retrospective cohort study was conducted on 106 patients undergoing HD. Demographic, clinical, and laboratory parameters were obtained three months after the effects of HD initiation was reviewed. **Results:** During a median follow-up of 24.5 months, 29 patients (27.3 percent) died. Non-survivors were significantly older (65.3 vs. 52.5 years, *p* < 0.001), had a higher prevalence of coronary artery disease (31 percent vs. 2.6 percent, *p* < 0.001), or shorter dialysis duration (14 vs. 27 months, *p* < 0.001). They also showed lower hemoglobin (9.2 vs. 10.1 g/dL, *p* = 0.007), creatinine (5.3 vs. 6.3 mg/dL, *p* = 0.048), and albumin levels (28 vs. 34 g/L, *p* = 0.001), as well as a higher MLR (0.329 vs. 0.254, *p* = 0.014). In multivariate analysis, age, CAR, and NLR independently predicted mortality, explaining 83.8% of the variation. ROC analysis identified age and MLR as significant predictors, with MLR showing a high negative predictive value (83.9%). The CALLY index did not demonstrate independent prognostic value. **Conclusions:** Age, CAR, NLR, and MLR were independent predictors of mortality in HD patients, whereas the CALLY index was not prognostic in this cohort. Among these markers, MLR may be a practical biomarker with strong negative predictive power. Larger prospective studies are needed to validate these findings.

## 1. Introduction

Chronic kidney disease (CKD) is defined as a form of structural and/or functional kidney damage that has persisted for at least three months [1]. CKD leads to uremia, oxidative stress, infections, dyslipidemia, malnutrition, hypervolemia, dialysis, periodontal diseases, and increased inflammation [2]. Systemic inflammation in end-stage kidney disease (ESKD) is a well-known risk factor for increased mortality in these patients and leads to muscle wasting, vascular calcification, depression, osteoporosis, and frailty [3].

ESKD patients often have poor nutrition; this leads to a decrease in protein and energy reserves, muscle loss, and increased frailty [4]. The presence of malnutrition is a significant predictor of morbidity and mortality in patients with ESKD [5]. Inflammation and malnutrition are complex in CKD/ESKD patients, leading to anorexia and increased fatigue and muscle breakdown, which contributes to muscle atrophy [4,6]. Recent studies have shown that patients receiving hemodialysis (HD) experience a decline in nutritional parameters and a high mortality rate due to malnutrition and inflammation [4,7]. Hypoalbuminemia has been identified as a significant factor associated with an increased risk of mortality, particularly among patients receiving dialysis treatment [8].

Immunometabolic dysregulation is a multifaceted condition involving various cell types, including T cells, B cells, macrophages, and dendritic cells. It is also associated with cytokines, chemokines, and metabolic processes such as oxidative stress, mitochondrial dysfunction, and inflammation [9,10]. Dysregulation of immune system cells and metabolism negatively impacts HD patients’ morbidity and mortality. In a recent study, a model incorporating neutrophil-to-lymphocyte ratio (NLR), monocyte-to-lymphocyte ratio (MLR), and platelet-to-lymphocyte ratio (PLR)-based inflammation scoring systems was employed by Liao et al., and a higher inflammation score was shown to be associated with all-cause mortality in HD patients. Thus, the development of biochemical markers reflecting inflammation, immune status, and nutrition, along with the assessment of HD patients’ prognosis, is essential [11].

The long-term exposure of CKD/ESKD and dialysis patients to chronic inflammation, nutritional deficiencies, and dysregulation of the immune system led to the hypothesis that the C-reactive protein–albumin–lymphocyte (CALLY) index score, which is the only marker combining these three pathogenic mechanisms of serum CRP, albumin, and lymphocyte count, could serve as a prognostic tool for mortality in dialysis patients. Incorporating biomarkers like the CALLY index into routine practice may enable precision nephrology by providing patient-specific risk profiles that support individualized treatment intensity, targeted nutritional and anti-inflammatory interventions, and informed modality selection. In our study, we aimed to investigate the role of inflammation, the immune system, and nutritional markers in predicting mortality in hemodialysis patients, focusing on the predictive performance of the C-reactive protein–albumin ratio (CAR), NLR, MLR, and the novel CALLY index (CRP, albumin, and lymphocyte index).

## 2. Materials and Methods

### 2.1. Patient Selection Criteria and Study Design

This retrospective cohort study was conducted at the SBU Gazi Yasargil Training and Research Hospital, enrolling HD patients between 1 January 2018 and 31 December 2023. The present study followed the principles of the Declaration of Helsinki. The research was approved by Health Sciences University, Diyarbakır Gazi Yaşargil Training and Research Hospital Local Ethics Committee, ethical approval code: 2025-317, approval date: 17 January 2025.

The study was conducted with the inclusion of patients aged over 18 years old who had undergone HD treatment for a minimum of three months. We included 106 patients who had been on dialysis. Patients lacking demographic and clinical data, receiving a kidney transplant, patients with concurrent malign tumors, and patients suffering from acute infections were not included in this study.

### 2.2. Data Collection and Laboratory Assessment

Demographic and clinical data were obtained from electronic medical records, encompassing various parameters, including age, gender, CKD etiology; diabetes mellitus (DM), hypertension (HT), autosomal dominant polycystic kidney disease, obstructive disease, glomerulonephritis, hereditary, monoclonal gammopathy of undetermined significance and coronary artery disease (CAD), dialysis duration, dialysis type, mortality, and relevant laboratory data. It is important to note that the collection of laboratory data was taken for the first time after 3 months of stable HD treatment, with exclusions made for cases of acute infection.

A comprehensive panel of biochemical variables was assessed, including hemoglobin, lymphocytes, neutrophils, platelets, monocytes, C-reactive protein (CRP), albumin, CRP/albumin (CAR), glucose, Blood Urea Nitrogen (BUN), creatinine, sodium, potassium, calcium, phosphorus, uric acid, and the NLR, MLR, PLR, and CALLY index. CALLY index = [albumin(g/L) × lymphocytes(109/L)/[C-reactive protein(mg/L) × 10] [12]. NLR, MLR, and PLR were calculated by dividing the number of monocytes and platelets, respectively, by the number of lymphocytes.

### 2.3. Statistical Analyses

The research data were computerized and evaluated using IBM SPSS 22 (IBM Statistical Package for Social Sciences). The descriptive statistics of the categorical variables were presented as numbers and percentages. The normal distribution of numerical variables was evaluated using the Kolmogorov–Smirnov and Shapiro–Wilk tests. The descriptive statistics of the categorical variables are presented as both absolute numbers and percentages. For the comparison of categorical variables, Pearson’s Chi-square test and Fisher’s exact test were applied using cross-tabulations. To ensure the validity of the results, a statistical technique known as the “Bonferroni Correction” was employed to manage multiple comparisons made within the subgroup tests. In the case of normally distributed numerical variables, mean values and standard deviations were reported as descriptive statistics. Conversely, numerical variables that were not normally distributed were reported as median values (minimum–maximum) in addition to their descriptive statistics. In the two-group analysis, an independent samples *t*-test was utilized to compare numerical variables with a normal distribution. In the context of a two-group analysis, the “Mann–Whitney U” test was employed to undertake a comparative analysis of numerical variables that were found to be non-normal. The independent risk factors affecting mortality were determined by means of Cox’s proportional hazards regression analysis. Independent variables with *p*-values less than 0.250 were included in the univariate analysis. In the multivariate Cox proportional hazards regression analysis, the CALLY index, CRP, albumin, and lymphocytes were included in two separate models due to their correlation with each other. Receiver-operating characteristic (ROC) analysis was employed to predict mortality. The independent variables that were used to formulate this prediction included sensitivity, specificity, positive predictive value, and negative predictive value. Statistical significance levels were accepted as *p* < 0.05 and interpreted as *p* < 0.001.

## 3. Results

The clinical characteristics of patients based on mortality status are presented in Table 1. The median age of the entire patient population was 58 years, and the proportion of female patients was 39.6% (n = 42). With respect to the underlying causes of CKD, DM was the primary etiological factor, accounting for 42.5% of cases, followed by HT, which accounted for 34%. The median follow-up period on dialysis was 24.5 months, with a minimum follow-up period of 4 months and a maximum period of 67 months. The mean age of patients who died was significantly higher than the age of patients who survived (65.3 vs. 52.5 years, *p* < 0.001). No statistically significant differences were found regarding the etiology of CKD between the study groups (*p* = 0.210). The rate of CAD was significantly higher in the non-survivor group compared to the survivor group (31% vs. 2.6, *p* < 0.001). The duration of time on HD was significantly shorter in the non-survivor group compared to the survivor group (14 months vs. 27 months, *p* < 0.001). Hemoglobin levels were significantly lower in the non-survivor group (9.2 g/dL) compared to the survivor group (10.1 g/dL, *p* = 0.007). Similarly, creatinine levels were also lower in the non-survivor group (5.3 mg/dL vs. 6.3 mg/dL, *p* = 0.048). Additionally, albumin levels were lower in the non-survivor group (28 g/dL) compared to the survivor group (34 g/dL, *p* = 0.001). The MLR was significantly higher in the non-survivor group (0.329 vs. 0.254, *p* = 0.014).

**Figure 1:**
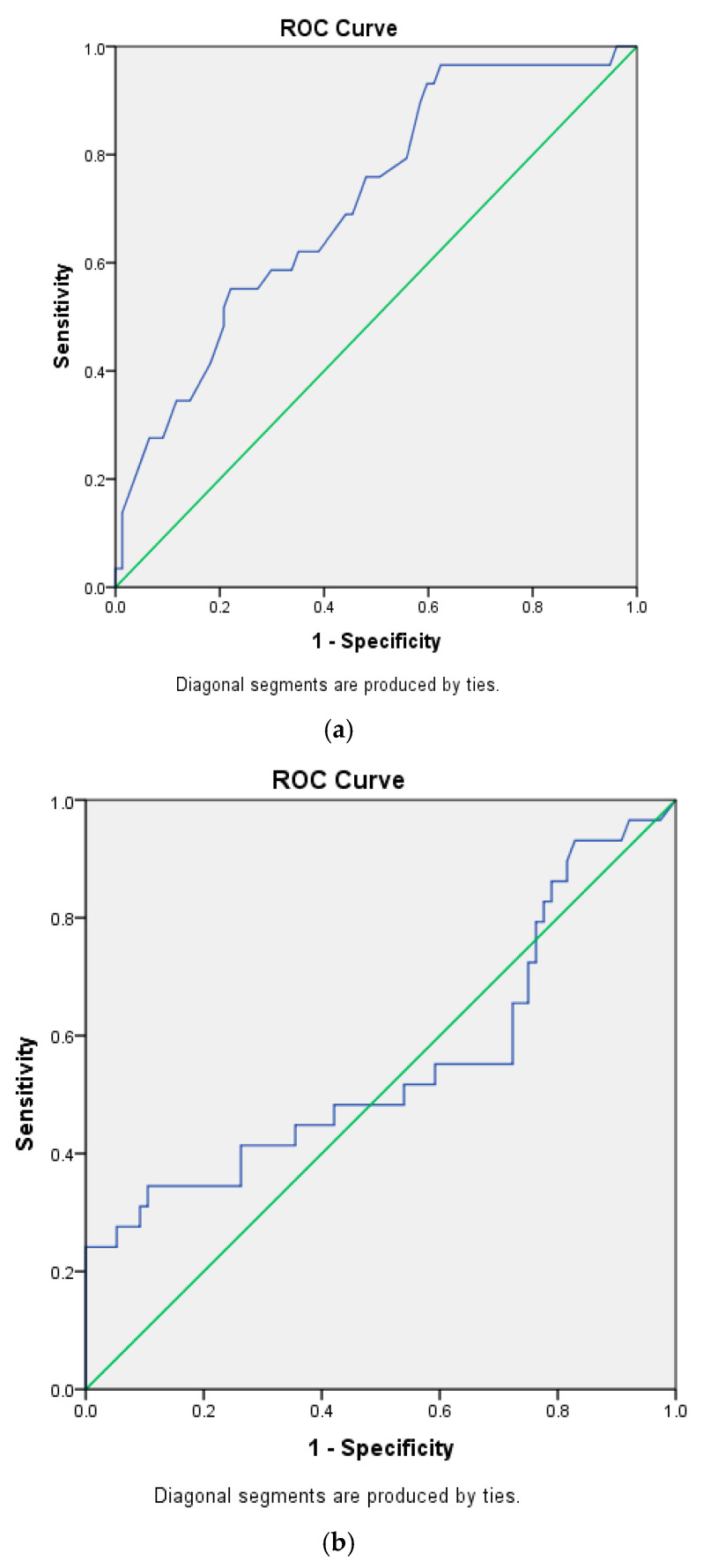

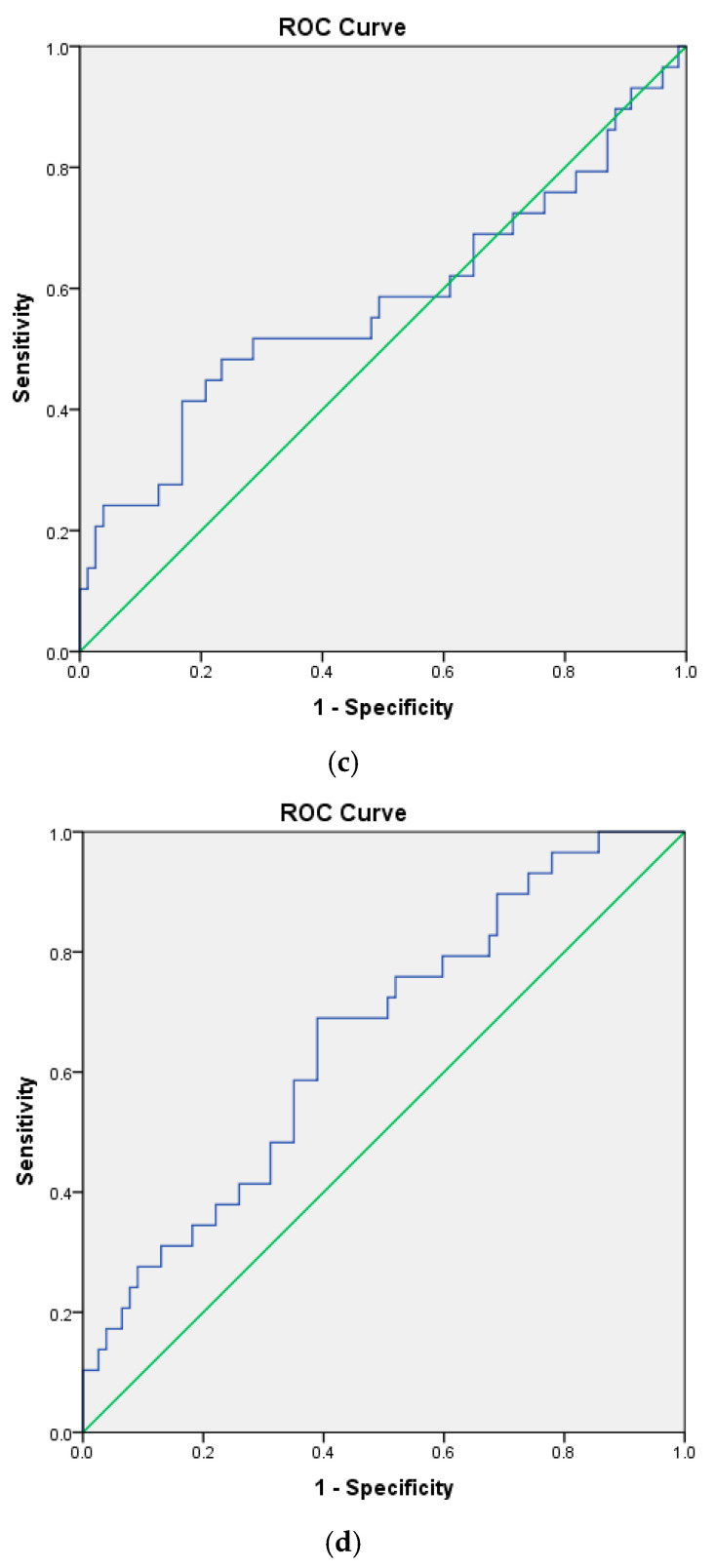
(**a**). Receiver-operating curve for age. (**b**). Receiver-operating curve for CRP/albumin. (**c**). Receiver-operating curve for NLR. (**d**). Receiver-operating curve for MLR. Blue for ROC curve, green for diagonal reference line.

Cox proportional hazards regression was employed to evaluate risk factors associated with mortality. The results of univariate and multivariate analyses are summarized in Table 2 and Table 3. In the multivariate models, Model 4 was identified as the optimal model, in which age, the CRP/albumin ratio, and the neutrophil/lymphocyte ratio accounted for 83.75% of the variation in mortality. In this model, each one-year increase in age, each unit increase in the CRP/albumin ratio, and each unit increase in the neutrophil/lymphocyte ratio were associated with 1.033-, 1.114-, and 1.049-fold increases in mortality, respectively. The ROC analysis results for the independent variables predicting mortality—including age, CRP/albumin ratio, neutrophil/lymphocyte ratio, and monocyte/lymphocyte ratio—are summarized in Table 4, with the corresponding ROC curves presented in Figure 1a–d.

ROC analysis identified age and the monocyte/lymphocyte ratio as significant independent predictors of mortality. Age was found to predict mortality with 62.1% sensitivity, 64.9% specificity, 39.5% positive predictive values (PPVs), and 61.5% negative predictive values (NPVs) at a cutoff of 61.5 years, while the monocyte/lymphocyte ratio predicted mortality with 69% sensitivity, 61% specificity, 40% PPVs, and 83.9% NPVs at a cutoff of 0.2866. Overall, both variables demonstrated greater predictive power for the absence of mortality (NPV) than for its occurrence (PPV).

## 4. Discussion

In this study, we evaluated inflammation, the immune system, and nutritional markers that were considered to be associated with all-cause mortality in hemodialysis patients. A predictive model including age, CAR, and NLR identified mortality with an accuracy of 83.75%. Nevertheless, the newly proposed prognostic index, CALLY, was not superior to conventional inflammation and immune system markers for independently predicting mortality.

DM was the primary etiological factor of CKD in our population, and the prevalence of patients with CAD was notably higher in the non-survivor group. The most common causes of kidney failure across the world are DM and cardiovascular disease, which are the leading causes of morbidity and mortality in CKD patients [13].

In our study, non-survivors were significantly older (mean age: 65.3 years) than survivors (mean age: 52.5 years), with age identified as a predictor of death. This supports the findings of previous studies [14,15]. This observation can be attributed to a confluence of factors in aging dialysis patients, including a higher burden of comorbidities, diminished physiological reserve, malnutrition, uremia and inflammation, an attenuated immune system, impaired executive function and neurophysiological activity, and an increased cardiovascular load [16,17].

Inflammation, immune dysfunction, and malnutrition are interrelated processes that frequently coexist in HD patients. Chronic inflammation in HD can impair immune competence, while malnutrition itself further exacerbates immune deficiency and predisposes to infections. Protein–energy wasting (PEW) is a frequent complication in this population and has been associated with increased mortality [18]. Persistent exposure to uremic toxins and systemic inflammation accelerates PEW by reducing oral intake, promoting catabolism, and contributing to muscle wasting [19]. Serum albumin, the most abundant protein synthesized in the liver, is a well-established marker of both nutritional and inflammatory status [20]. Beyond maintaining plasma oncotic pressure, albumin exerts antioxidant effects by scavenging free radicals and contributes to vascular permeability and acid–base homeostasis [20]. Hypoalbuminemia may result from malnutrition in the general population, but in CKD patients, it often reflects a combination of reduced nutrient intake, chronic inflammation, increased catabolism, impaired anabolism, and dialysis-associated protein loss [21]. In a recent study by Lai KJ et al., lower serum albumin levels were independently associated with higher mortality rates in dialysis patients [22]. In line with the previous literature, our findings demonstrated that HD patients who died had significantly lower albumin levels compared with survivors. In addition, our multivariate analyses showed that the C-reactive protein-to-albumin ratio was a strong and independent predictor of mortality. Recent data from a cohort of 787 HD patients indicated that an elevated CAR was significantly associated with increased mortality risk within the first six months of dialysis [23]. Another study also supported CAR as a reliable prognostic biomarker in this setting [24]. The observed relationship between CAR and adverse outcomes in ESKD is likely attributable to the combined effects of chronic inflammation, malnutrition, and accelerated atherosclerosis. It is noteworthy that up to half of HD patients develop a protein–energy deficiency. The underlying mechanisms behind this are multifactorial and include poor dietary intake, dietary restrictions, chronic inflammation, oxidative stress, nutrient loss through urine or dialysate, decreased anabolic hormones, increased catabolic drive, metabolic acidosis, sarcopenia, and age-related functional decline [25]. These overlapping pathways reinforce the clinical relevance of nutritional and inflammatory markers such as albumin and CAR in the risk stratification of HD patients.

In CKD, inflammation contributes to complications through several mechanisms, including increased skeletal muscle protein degradation, reduced muscle protein synthesis, and enhanced insulin resistance [26]. Atherosclerosis is a frequent complication in ESKD and is driven by inflammation, malnutrition, and heightened oxidative stress [27]. These processes result in abnormalities in lipid and lipoprotein metabolism as well as endothelial dysfunction. HD patients with elevated serum CRP and reduced albumin levels are often described as having malnutrition–inflammation–atherosclerosis syndrome, which highlights the association between these factors and the progression of advanced atherosclerotic cardiovascular disease [28]. Given that both inflammation and malnutrition are independently associated with increased mortality, the CAR serves as a valuable biomarker for assessing the combined impact of malnutrition and inflammation on mortality risk in HD patients. In our study, the combination of CRP and albumin expressed as CAR provided a more sensitive predictor of mortality than either CRP or albumin alone.

In our study, we identified the NLR as a strong and independent predictor of mortality in HD patients. The NLR is a well-recognized biomarker of systemic inflammation [29]. Neutrophils play a crucial role in innate immune defense and exert regulatory effects on adaptive immunity, serving as the principal effector cells in systemic inflammatory response syndrome [29]. Lymphocytes, another key component of the immune system, are responsible for both protection against external infections and recognition within the immune system [30]. Thus, the NLR integrates the innate immune response, largely mediated by neutrophils, with the adaptive immune response, mediated by lymphocytes [31]. Several studies have demonstrated that an elevated NLR is an independent risk factor for mortality in HD patients [29,32]. Consistent with these findings, our results confirmed the NLR as a significant predictor of mortality. As an inexpensive and easily measurable marker of systemic inflammation, the NLR reflects both the intensity of a patient’s pro-inflammatory state, through increased neutrophil counts, and the degree of immunosuppression, through reduced lymphocyte counts. Although we were unable to establish a clear cutoff value based on ROC curve analysis, multivariate analyses revealed that higher NLR values were significantly associated with increased mortality.

In our study, higher MLR values were associated with an increased risk of death, whereas lower MLR values strongly predicted improved survival. MLR is a novel biomarker that, similar to NLR, reflects both innate and adaptive immune pathways and can be easily calculated from routine blood parameters [33]. Recent studies have also demonstrated that MLR is a strong predictor of cardiovascular mortality in patients undergoing HD and peritoneal dialysis [34,35]. In our cohort, elevated MLR likely reflected a predominance of pro-inflammatory responses accompanied by a reduced immunosuppressive capacity within the immune system. These findings suggest that MLR may serve as a valuable biomarker for mortality prediction in HD patients. Notably, the high negative predictive value observed in our study indicates that patients with lower MLR values are more likely to have favorable survival outcomes.

The CALLY index, derived from serum CRP, albumin, and lymphocyte count, is a novel biomarker that reflects inflammation, nutritional status, and immune function. Previous studies have demonstrated its prognostic value as an independent factor in various malignancies [36,37]. Recently, Huang J. et al. reported for the first time, through multivariate analysis, a correlation between the CALLY index and all-cause mortality in HD patients [14]. In our study, we compared the CALLY index between survivors and non-survivors; however, we did not observe a significant difference between the two groups. Although the CALLY index has emerged as a prognostic indicator in the context of malignancy and inflammatory diseases, this association was not evident in our cohort. This discrepancy may be explained by the relatively small sample size, the limited follow-up duration, or the possibility that the prognostic strength of the CALLY index is restricted in the CKD population. Therefore, large-scale prospective studies are warranted to clarify the prognostic utility of the CALLY index in predicting mortality among patients with renal failure.

However, this study has several limitations. First, it was a retrospective analysis of a relatively small sample size. Second, we only collected CRP, lymphocyte count, and albumin levels on the third month after starting dialysis, without analyzing any dynamic changes or their impact on prognosis.

## 5. Conclusions

In this study, we compared inflammation, the immune system, and nutritional markers affecting mortality in hemodialysis (HD) patients. Using a model consisting of age, CAR, and NLR, we predicted mortality with an accuracy of 83.75%. We found that the novel biomarker CALLY index was not superior to traditional markers in HD patients. The high negative predictive value of MLR indicated that patients with low MLR had better survival outcomes. In conclusion, CAR, NLR, and MLR emerged as reliable and practical markers for predicting mortality in HD patients, whereas the prognostic value of the CALLY index requires validation in larger, prospective studies.

## Figures and Tables

**Table 1:** Clinical characteristics of patients by mortality status.

	All Patients (n = 106)	Survivors (n = 77)	Non-Survivors (n = 29)	*p*
Age	58 (18–91)	52.5 ± 16.7	65.3 ± 14.7	<0.001 ^a^
Gender				0.501 ^b^
Female	42 (39.6%)	29 (37.7%)	13 (44.8%)	
Male	64 (60.4%)	48 (62.3%)	16 (55.2%)	
CKD etiology				0.210 ^b^
DM	45 (42.5%) ^a^	28 (36.4%)	17 (58.6%)	
HT	36 (34%) ^a^	31 (40.3%)	5 (17.2%)	
ADPCD	3 (2.8%) ^a^	3 (3.9%)	0 (0%)	
Obstructive diseases	3 (2.8%) ^a^	2 (2.6%)	1 (3.4%)	
Glomerulonephritis	2 (1.9%) ^a^	2 (2.6%)	0 (0%)	
Hereditary	1 (0.6%) ^a^	1 (1.3%)	0 (0%)	
MGUS	1 (0.9%) ^a^	1 (1.3%)	0 (0%)	
Unknown	15 (14.2%) ^a^	9 (11.7%)	6 (20.7%)	
DM, y/n (y%)	49/57 (46.2%)	45/32 (41.6%)	12/17 (58.6%)	0.116 ^b^
HT, y/n (y%)	72/34 (67.9%)	25/52 (67.5%)	9/20 (69%)	0.888 ^b^
CAD, y/n (y%)	11/95 (10.4%)	75/2 (2.6%)	20/9 (31%)	<0.001 ^c^
Dialysis duration, months	24.5 (4–67)	27 (8–65)	14 (4–67)	<0.001 ^d^
Neutrophil count, µL	4930 (760–17,750)	4950 (1620–10,120)	4840 (760–17,750)	0.697 ^d^
Hemoglobin, g/dL	9.9 ± 1.6	10.1 ± 1.5	9.2 ± 1.7	0.007 ^a^
Lymphocyte count, µL	1560 (250–4500)	1610 (610–4500)	1450 (250–3930)	0.082 ^d^
Monocyte count, µL	469 ± 190	455 ± 190	506 ± 189	0.222 ^a^
Platelet, 10^3^/µL	210 (75–662)	217 (75–447)	181 (88–662)	0.294 ^d^
Glucose, mg/dL	121 (50–626)	115 (50–626)	132 (78–613)	0.573 ^d^
BUN, mg/dL	115 ± 41	113 ± 40	120 ± 43	0.544 ^a^
Creatinine, mg/dL	6.12 (1.1–14.5)	6.3 (1.1–14.5)	5.3 (2.3–9.3)	0.048 ^d^
Uric acid, mg/dL	6.1 (2.1–11.5)	6.3 ± 1.6	5.8 ± 1.4	0.142 ^a^
CRP, mg/L	6.2 (2–237.2)	6.3 (2–73.6)	5.2 (2–237.2)	0.732 ^d^
Albumin, g/L	33 (7–45)	34 ± 4.4	28 ± 8.3	0.001 ^a^
CRP/albumin ratio	0.203 (0.05–33.89)	0.205(0.05–2.63)	0.179 (0.05–33.89)	0.410 ^d^
NLR	2.94 (0.74–71)	2.81 (0.74–10.8)	3.59 (0.92–71)	0.230 ^d^
MLR	0.271 (0.06–2.08)	0.254 (0.06–0.66)	0.329 (0.14–2.08)	0.014 ^d^
PLR	0.129 (0.04–0.60)	0.122 (0.04–0.36)	0.157 (0.05–0.60)	0.107 ^d^
Sodium, mmol/L	138 (128–164)	138 (128–164)	137 (128–147)	0.316 ^d^
Potassium, mmol/L	4.6 ± 0.8	4.5 ± 0.8	4.6 ± 1	0.634 ^a^
Calcium, mmol/L	8.2 ± 0.7	8.3 ± 0.6	8.1 ± 0.8	0.369 ^a^
Phosphorus, mmol/L	4.6 (1.5–9.6)	4.5 (1.5–9.6)	4.9 (2–9.2)	0.845 ^d^
CALLY index	0.804 (0.004–6.46)	0.803 (0.064–6.46)	0.898 (0.004–4.258)	0.231 ^d^

^a^ Independent sample-t. ^b^ Pearson Chi-square test. ^c^ Fisher’s exact test. ^d^ Mann–Whitney U test. CKD: Chronic Kidney Disease; DM: Diabetes Mellitus; HT: Hypertension; ADPCD: Autosomal Dominant Polycystic Kidney Disease; MGUS: Monoclonal Gammopathy of Undetermined Significance; CAD: Coronary Artery Disease; BUN: Blood Urea Nitrogen; CRP: C-Reactive Protein; NLR: Neutrophil-to-Lymphocyte Ratio; MLR: Monocyte-to-Lymphocyte Ratio; PLR: Platelet-to-Lymphocyte Ratio; CALLY: C-Reactive Protein–Albumin–Lymphocyte; y: Yes; n: No.

**Table 2:** Risk factors affecting mortality.

Cox Proportional Hazards Regression	Univariate		
	CI (95%)	HR	*p*
Age	1.013–1.069	1.041	0.004
DM, y/n (y%)	0.698–3.120	1.475	0.309
HT, y/n (y%)	0.381–1.866	0.844	0.675
CAD, y/n (y%)	1.840–9.595	4.201	0.001
Neutrophil count, µL	1.00–1.00	1.00	0.063
Monocyte count, µL	0.999–1.003	1.001	0.529
Hemoglobin, g/dL	0.501–0.844	0.651	0.001
Lymphocyte count, µL	0.999–1.000	0.999	0.074
NLR	1.021–1.081	1.051	0.001
MLR	2.411–18.117	6.609	<0.001
PLR	2.607–5201.1	116.440	0.014
BUN, mg/dL	0.995–1.013	1.004	0.416
Creatinine, mg/dL	0.748–1.024	0.875	0.095
Uric acid, mg/dL	0.691–1.113	0.877	0.281
CRP, mg/L	1.009–1.020	1.014	<0.001
Albumin, g/L	0.157–0.459	0.268	<0.001
CRP/albumin ratio	1.103–1.265	1.181	<0.001
Calcium, mmol/L	0.505–1.498	0.870	0.616
Phosphorus, mmol/L	0.835–1.533	1.131	0.427
CALLY index	0.670–1.226	0.907	0.525

DM: Diabetes Mellitus; HT: Hypertension; CAD: Coronary Artery Disease; BUN: Blood Urea Nitrogen; CRP: C-Reactive Protein; NLR: Neutrophil-to-Lymphocyte Ratio; MLR: Monocyte-to-Lymphocyte Ratio; PLR: Platelet-to-Lymphocyte Ratio; CALLY: C-Reactive Protein–Albumin–Lymphocyte; y: Yes; n: No.

**Table 3:** Predictive models for mortality risk.

Model	R^2^	Age	CAD	CRP/Albumin Ratio (CAR)	NLR	MLR	PLR
		*p* HR CI 95%	*p* HR CI 95%	*p* HR CI 95%	*p* HR CI 95%	*p* HR CI 95%	*p* HR CI 95%
1	58.7%	0.039 1.030 (1.001–1.059)					
2	76.8%	0.039 1.030 (1.002–1.059)		<0.001 1.139 (1.060–1.224)			
3	65.4%	0.028 1.033 (1.003–1.063)			0.013 1.042 (1.009–1.076)		
4	83.75%	0.026 1.033 (1.004–1.064)		0.003 1.114 (1.038–1.195)	0.004 1.049 (1.015–1.083)		
5	61.2%	0.033 1.033 (1.003–1.064)				0.014 4.154 (1.327–13.002)	
6	80.2%	0.034 1.032 (1.002–1.063)		0.001 1.127 (1.052–1.207)		0.009 5.078 (1.495–17.225)	
7	56.3%	0.035 1.031 (1.002–1.061)					
8	74.8%	0.029 1.032 (1.003–1.062)		0.002 1.122 (1.042–1.206)			

CAD: Coronary Artery Disease; NLR: Neutrophil-to-Lymphocyte Ratio; MLR: Monocyte-to-Lymphocyte Ratio; PLR: Platelet-to-Lymphocyte Ratio; CAR: C-Reactive Protein–Albumin Ratio.

**Table 4:** ROC analysis results for age, CRP/albumin ratio, NLR, and MLR.

	AUC	CI 95%	Sensitivity	Specificity	PPV	NPV	Cut off Value	*p*
Age	0.712	0.605–0.820	62.1%	64.9%	39.5%	80.9%	61.5	0.001
CRP/Albumin Ratio (CAR)	0.552	0.415–0.690	–	–	–	–	–	0.410
NLR	0.576	0.439–0.713	–	–	–	–	–	0.230
MLR	0.656	0.542–0.769	69%	61%	40%	83.9%	0.2866	0.014

ROC: receiver-operating curve; NLR: neutrophil-to-lymphocyte ratio; MLR: monocyte-to-lymphocyte ratio.

## Data Availability

The datasets used and/or analyzed during the current study are available from the corresponding author on reasonable request.
